# Leader’s Possession of Linguistic Intelligence in Relation to Leader–Member Exchange Theory

**DOI:** 10.3390/jintelligence11050092

**Published:** 2023-05-12

**Authors:** Timotej Ribič, Miha Marič

**Affiliations:** 1 Ribič Sašo s.p., Zgornje Pirniče 123, 1215 Medvode, Slovenia; timotej.ribic@student.um.si; 2 Faculty of Organizational Sciences, University of Maribor, Kidričeva ulica 55 A, 4000 Kranj, Slovenia

**Keywords:** multiple intelligences, linguistic intelligence, communication, relationships, leadership, LMX, human resource management, organizational behavior

## Abstract

When practicing high-quality leader–member exchange (LMX) theory, the leader’s ability to communicate, build and maintain relationships is a vital part. Because leader–member exchange theory is a relationship-based approach to leadership that primarily includes social exchange and communication on a daily basis, we can highlight linguistic intelligence as a key leadership skill that is part of the multiple intelligences defined by Howard Gardner. The goal of this article was to conduct research into organizations where the leader applies LMX theory and examine whether the leader’s linguistic intelligence is positively related to the quality of the leader–member exchange. The dependent variable was the quality of the LMX. We were able to recruit 39 employees and 13 leaders. Correlations and multiple regressions were used to analyze our statement. The overall results are statistically significant and we conclude that there is a high positive correlation between LMX and linguistic intelligence in the organizations that were part of this study. A limitation of this study is the use of purposive sampling, which resulted in a relatively small sample size and may limit the generalization of the results to other populations.

## 1. Introduction

In the business environment, there are several well-known leadership styles and approaches used in various organizations. Leadership represents a key element of management since one of the most important functions of a leader is to realize the organization’s vision based on achieving goals through directing, influencing and motivating employees (Juneja 2021).

Among the more innovative but less implemented approaches to leadership is the so-called leader–member exchange theory, or LMX, which first appeared in the 1970s (Stone 2017). LMX theory is based on the social exchange and relationship between a leader and his or her member or employee (Graen and Uhl-Bien 1995). The focus is on adapting the leader’s approach to each employee individually, with the central goal of recognizing and building quality mutual relationships and social exchanges through communication between the leader and the employee on a day-to-day basis (Erdogan and Bauer 2015; Hirvi et al. 2021). Organizations that adopt this type of leadership approach show a better organizational climate, greater trust and mutual respect between the leader and employees, greater employee independence and dedication to work and, in some cases, greater staff engagement and less turnover (Mohamad et al. 2019; Gerstner and Day 1997).

It is well known that LMX theory is based on the mutual relationships between a leader and an employee, in which communication plays an essential role (Northouse 2016). Therefore, we can highlight linguistic intelligence as a central skill of a leader, which is part of the multiple intelligences defined by Gardner (1983, 2010). Since much has been written and is known about LMX theory, the correlation between the leader’s possession of linguistic intelligence and the practice of quality LMX is less known and has been unexplored.

In this study, we proceed from the thesis that states the following: “The leader’s linguistic intelligence is positively related to the quality of the Leader–member exchange”.

### 1.1. Leader–Member Exchange Theory

Leader–member exchange theory or LMX is a relationship-based approach to leadership that focuses on the relationship between the leader and his or her followers or employees (Graen and Uhl-Bien 1995). In addition to mutual relations, LMX theory primarily includes social exchange and communication between the leader and the employee on a day-to-day basis (Hirvi et al. 2021).

LMX theory advocates that leadership consists of developing a two-way, person to person relationship between the leader and the employees to ensure personal development and growth within the organization (Janse 2019). The quality of such a relationship between the leader and the member is measured by the level of trust, respect and support, and the loyalty of employees (Shaikh et al. 2019).

In addition, leaders do not treat all employees equally, but rather adapt their approach to each employee individually with the central awareness that employees are unequal among themselves (Dunaetz 2020). The way that leaders adapt their approach and communication ensures building good relationships, good communication and mutual respect in person to person interactions, which consequently translates into a better organizational climate, higher work engagement, bigger commitment and less employee turnover (Leadem 2018; Dunaetz 2020; Niswaty et al. 2021; Janse 2019; Graen and Uhl-Bien 1995).

Among all leadership styles and approaches, LMX theory stands out in terms of focusing on employees and caring for their well-being, which in turn contributes to greater work efficiency (Erdogan and Bauer 2015). Previous research has also established that the practice of quality LMX is positively related to employee performance, overall satisfaction, commitment, conflict resolution and lower turnover in the organization (Gerstner and Day 1997; Dunaetz 2020; Niswaty et al. 2021).

Through the use of LMX and through communication, the leader shows greater care and trust toward employees, which contributes to higher and closer employee cooperation. More importantly, when LMX is interpreted as a positive norm among employees, they are more likely to demonstrate stronger work engagement (Wagner and Koob 2022; Decuypere and Schaufeli 2020).

LMX theory is considered to be multidimensional, meaning that it can be implemented in different levels of the organization (Hirvi et al. 2021). The quality of the mutual relationship between the leader and employee determines the quality of the LMX (Graen and Uhl-Bien 1995). In order to assess and measure the quality of the LMX in the organization and thus the quality of the mutual relationship between the leader and employee, the pioneers of LMX theory, Graen and Uhl-Bien (1995), designed a questionnaire called LMX-7, which shows a high degree of reliability. The LMX-7 questionnaire is considered the gold standard when researching the presence and quality of the LMX.

A leader is a person who positively influences the thoughts and behavior of the people around him or her, who is able to perform specific tasks and who has a high personal status within the organization (Bulturbayevich et al. 2021). Regardless of which leadership style is implemented in an organization, the leader must possess certain basic competencies and skills to practice quality leadership (Som et al. 2020). 

Within the framework of LMX theory, communication has been repeatedly mentioned by authors (Graen and Uhl-Bien 1995; Erdogan and Bauer 2015; Northouse 2016; Hirvi et al. 2021; Decuypere and Schaufeli 2020) as being a very important component in practicing and implementing quality LMX. Accordingly, we have highlighted linguistic intelligence as defined by Gardner (1983, 2010) as a central skill of a leader who implements and practices LMX within the organization.

### 1.2. Linguistic Intelligence

In 1983, Harvard University psychologist and professor Howard Gardner challenged the theory that human intelligence is a unique concept which can be measured by IQ tests, stating that this is too limited (Gardner 1983). Instead, Gardner (1983) suggested that human intelligence consists of eight distinct intelligences, each representing a different range of human ability and capability to process information.

One of the eight intelligences is called “Linguistic intelligence”, sometimes called “Language intelligence” (Gardner 1983, 2010; Fambro 2019). This intelligence includes a person’s enhanced ability to use and understand words efficiently, and their ability to recognize the sound and rhythm of pronunciation, order among words and their meaning (Cherry 2022). It also includes the clarity of speech and the richness of a person’s vocabulary (Burgov 2015). Persons with a high level of developed linguistic intelligence are usually excellent at reading, writing stories, memorizing words and giving speeches, and also show higher-order critical thinking skills (Setiawan et al. 2020).

Although linguistic intelligence is usually considered to be the most important skill of writers and poets, it is also a means by which we can create and be a part of social interactions with others (Georgieva 2020). In this context, linguistic intelligence is a skill that every person possesses from birth. It differs only in the degree of development in individuals and the environment in which it can be supported and used (Islam 2019; Kurniaman et al. 2020).

In summary, linguistic intelligence involves a person’s enhanced ability to operate with words, both orally and in writing, and involves breaking down sentences and interpreting what is said and heard with associated emotional expressions of the individual that can be perceived through social exchanges (Gardner 1983; Georgieva 2020; Cherry 2022).

At the organizational level, a leader who possesses developed linguistic intelligence can have the ability to communicate with employees in a high-quality manner, calm conflicts by choosing appropriate words and transfer knowledge well, and can identify both employee satisfaction and dissatisfaction by interpreting what is heard (Setiawan et al. 2020; Cherry 2022; Niswaty et al. 2021).

To identify the extent to which an individual possesses or has developed linguistic intelligence, this intelligence can be measured and evaluated using a questionnaire called CUIM (Aliaga et al. 2014), which is based on the definition of linguistic intelligence developed by Gardner (1983).

## 2. Materials and Methods

For the research part, we used the strategy of a quantitative research method using online questionnaires, where the research units were leaders who practice LMX in medium-sized organizations and employees who are under the influence of LMX theory in the same organizations. We researched the field of leadership and studied the leader and employees based on a humanistic approach. The research methodology was thus based on the principles of a case study, as we investigated a complex social phenomenon in the field of business and leadership (Yin 2009).

Based on public information and actual observation in the field, with physical access to the organizations, we were able to carry out purposive sampling and selected 3 organizations who demonstrated practicing LMX on a day-to-day basis (Ribič and Marič 2021), and were able to recruit 39 employees and 13 leaders who were either project or department leaders.

The research was conducted by submitting formed questionnaires to the leaders and employees in the mentioned organizations. The quality of the mutual relationship between the leaders and the employees, and thus the quality of the leader–member exchange, was measured and evaluated using the “LMX-7” questionnaire developed by Graen and Uhl-Bien (1995). The questionnaire contains 7 statements, and each of which can be answered on a 5-point Likert scale, where 1 is “never” and 5 is “always”. The questionnaire has been repeatedly used and validated and has an excellent level of reliability (George and Mallery 1995), with a Cronbach alpha coefficient value of α = 0.925. The questionnaire was filled out by employees.

To measure and evaluate linguistic intelligence, we used part of the CUIM questionnaire to measure multiple intelligences (Aliaga et al. 2014). In the first section of the CUIM questionnaire, ten questions are directly related to linguistic intelligence, as proposed by Gardner (1983). Each variable is measured using a 5-point Likert scale, where 1 is “strongly disagree” and 5 is “strongly agree”. The first section of the questionnaire shows an acceptable level of reliability (George and Mallery 1995), with a Cronbach alpha coefficient value of α = 0.75. The questionnaire was filled out by the leaders in the organizations.

In order to carry out the research in Slovenia and in the Slovenian business environment, both questionnaires were translated from English into Slovenian. The LMX-7 questionnaire can be found in Appendix A, and the linguistic intelligence questionnaire can be found in Appendix B.

After the research, the data obtained were processed and analyzed using IBM SPSS Statistic version 28.0.0 and Microsoft Office Excel version 2016 in terms of descriptive statistics, correlations were found using Pearson and Spearman’s correlation coefficient and regression analysis including ANOVA test was used to test our thesis that states the following: “The leader’s linguistic intelligence is positively related to the quality of the Leader-member exchange”.

## 3. Results

### 3.1. LMX-7 Questionnaire Data Validation

First, we validated the collected data. We checked whether there were any corrupted data or missing values among the collected data from the LMX-7 questionnaire. We performed data validation using data reliability analysis, as shown in Table 1, and came to the result that there were no missing values in LMX data. The validity and reliability of the obtained results are shown in Table 1 (a) using Cronbach’s alpha coefficient with a value of α = 0.831.

### 3.2. Linguistic Intelligence Questionnaire Data Validation

We also validated the collected data from the linguistic intelligence questionnaire. We checked whether there were any corrupted data or missing values among the collected data. We performed data validation using data reliability analysis, as seen in Table 2, and came to the result that there were no missing values for linguistic intelligence data. The validity and reliability of the obtained results are shown in Table 2a using a Cronbach alpha coefficient with a value of α = 0.617.

### 3.3. Questionnaire Score and Reliability Analysis for LMX Questionnaire

We examined the level of quality at which LMX actually exists in the organizations where we conducted the research. Following the LMX-7 scale, we added up the scores for each question, then averaged all of the sums and arrived at the result $\overset{-}{x}$ = 29.02, as shown in Table 3. Following the LMX-7 scale, we can interpret the quality of the LMX using the following guidelines: very high = 30–35, high = 25–29, moderate = 20–24, low = 15–19 and very low = 7–14. Scores in the upper ranges indicate stronger, higher-quality leader–member exchanges, whereas scores in the lower ranges indicate exchanges of lesser quality (Graen and Uhl-Bien 1995). From the result and the scale, we can conclude that there is a high quality of LMX in the organizations where we conducted the research.

Then, we performed an internal reliability test for the LMX questionnaire. Table 3 (a) shows that the LMX questionnaire, which was used to measure the quality of the leader–member exchange and was filled out by employees, shows good reliability with a Cronbach alpha coefficient of α = 0.831.

### 3.4. Questionnaire Score and Reliability Analysis for Linguistic Intelligence Questionnaire

The process was repeated for the linguistic intelligence questionnaire completed by the leaders. We conducted a comparison of means test based on the answers using a 5-point Likert scale (from 1 to 5) and obtained the results shown in Table 4. Table 4 (a) shows the average of the means from Table 4, which is $\overset{-}{x}$ = 4.03. We can conclude that the leaders included in the research have high linguistic intelligence.

Next, we performed an internal reliability test. As Table 5 shows, the linguistic intelligence questionnaire, which was used to measure the leader’s possession of linguistic intelligence, and was filled out by leaders, shows reliability with a Cronbach alpha coefficient of α = 0.617, which is still considered to be acceptable.

### 3.5. Descriptive Statistics for LMX Questionnaire

The results in Table 6 show that 39 employees of the organizations in which we conducted the research completed the questionnaire in full. Table 6 (a) shows that the questionnaire was completed by 13 (33.3%) females and 26 (66.7%) males.

The results in Table 6 (a) show that employees were on average $\overset{-}{x}$ = 35 years old with a standard deviation of s = 3.69. The youngest employee was 29 years old and the oldest was 43 years old.

The results in Table 6 (b) show a correlational test with which we checked the relationship between the age and gender of employees and the influence of this alone on the data of the LMX-7 questionnaire. Table 6 (b) shows that gender and age were not statistically significant for the gathered data and thus did not affect the results of the LMX-7 questionnaire, as shown by the values for LMX-7 and gender, r = 0.082 and *p* = 0.622, at a 5% level of significance of α = 0.05, where the *p*-value was higher than the significance level (*p* > α), and the values for LMX-7 and age, r = 0.257 and *p* = 0.114, at a 5% level of significance of α = 0.05, where the *p*-value was higher than the significance level (*p* > α).

### 3.6. Descriptive Statistics for Linguistic Intelligence Questionnaire

The results in Table 7 show the gender of the leaders in the organizations included in the research. The questionnaire was completed by 4 (30.8%) females and 9 (69.2%) males.

From Table 7 (a), we can see that the age of the leaders was somewhat scattered. The average age of the leaders was $\overset{-}{x}$ = 41 years, with a standard deviation of s = 11.52. The youngest leader was 26 years old and the oldest was 58 years old.

The results in Table 7 (b) show a correlational test with which we checked the relationship between the age and gender of leaders and the influence of this alone on the data of the linguistic intelligence questionnaire. Table 7 (b) shows that gender and age were not statistically significant for the gathered data and thus did not affect the results of the linguistic intelligence questionnaire, as shown by the values for linguistic intelligence and gender, r = −0.095 and *p* = 0.757, at a 5% level of significance of α = 0.05, where the *p*-value was higher than the significance level (*p* > α), and the values for linguistic intelligence and age, r = 0.322 and *p* = 0.283, at a 5% level of significance of α = 0.05, where the *p*-value was higher than the significance level (*p* > α).

### 3.7. Correlational Analysis

We performed a correlation test between LMX as our dependent variable and linguistic intelligence as our independent variable. As shown in Table 8, we used the Pearson correlation coefficient and concluded that there was a positive high correlation as shown by the values of r = 0.724 and *p* = 0.005, at a 1% level of significance of α = 0.01, where the *p*-value was lower than the significance level (*p* < α).

We also performed the Spearman correlation coefficient for the same variables, with LMX as our dependent variable and linguistic intelligence as our independent variable. As can be seen in Table 9, the Spearman correlation coefficient analysis came back with the result that there was a positive moderate correlation with the values of r = 0.580 and *p* = 0.038, at a 5% level of significance of α = 0.05, where the *p*-value was lower than the significance level (*p* < α).

### 3.8. Regression Analysis

Linear regression analysis was performed to test the impact of the independent variable (linguistic intelligence) on the dependent variable (LMX). The standardized beta value and *p* value were used to test whether our statements in the form of a thesis were supported or not.

The summary of the regression model in Table 10 shows us how well the model fits the data. The standard error of the estimate is measured in units of the response to the variable. In this case, the linguistic intelligence represents the standard difference in the data values that fall from the regression line. The standard error of the estimates is s = 1.36802. The more the equation predicts the response, the lower the value of standard error is.

R square describes the amount of variation observed in the response variable (LMX) explained by the predictor variable (linguistic intelligence). Table 10 shows that linguistic intelligence as a predictor caused 52.4% (r^2^ = 0.524) of the variation to the LMX response variable.

The ANOVA test in Table 11 shows the overall impact of the model. It depicts the amount of variation in the response data that is explained by the predictor (linguistic intelligence) and the amount of variation that remains unexplained. In our model, the *p* value in the ANOVA test is *p* = 0.005 (*p* < α), which shows that the results are statistically significant and that the predictor variable (linguistic intelligence) makes a significant contribution to the dependent variable (LMX).

Table 12 shows the coefficient result of linear regression. The coefficient table shows that the *p* value is *p* = 0.005 which is lower than the predetermined level of significance of α = 0.05 (*p* < α). We conclude that the results are statistically significant and that the data provide sufficient evidence to conclude that the leaders’ possession of linguistic intelligence has an impact on the quality of the leader–member exchange.

## 4. Discussion

Linking LMX with the leader’s linguistic intelligence was a major objective of this study. We wanted to find out whether the leader’s linguistic intelligence was a condition for the quality of practicing LMX. Today, LMX theory is considered a topic worthy of research. Leader–member exchange was introduced in the 1970s (Stone 2017) and is based on the potential of social exchange between the leader and the employee, including communication, relationship building and mutual respect (Graen and Uhl-Bien 1995).

LMX theory has received a lot of support from its inception, as organizations that adopt this type of leadership approach show a better organizational climate, greater trust and more respect between the leader and the employees (Mohamad et al. 2019; Gerstner and Day 1997). All of these things lead to employee well-being and have a positive effect on greater staff engagement, greater employee satisfaction, lower turnover and fewer safety incidents (Gallup 2015).

Day by day, organizations are placing more and more emphasis on good mutual relationships, and it is up to the leader to build and maintain such relationships. Therefore, communication is extremely important in LMX (Northouse 2016); so, we highlighted linguistic intelligence as a central skill of a leader (Gardner 1983, 2010), formed a corresponding thesis and tested it via further research.

We conducted a survey using an online questionnaire, which was distributed to three organizations that we selected based on purposive sampling, due to their demonstration of practicing LMX on a day-to-day basis (Ribič and Marič 2021). The research methodology was based on case study principles, namely, we were investigating a complex social phenomenon, and case study methodology is considered to be commonly used in the sciences of psychology, sociology, business and other fields (Yin 2009). Purposive sampling was used based on access to publicly known information about organizations and actual observation in the field, namely, we had physical access to these organizations.

We investigated the field of leadership and studied the leader and employees based on a humanistic approach, with the central goal of determining whether the leader’s linguistic intelligence had an impact on the quality of the LMX.

We managed to obtain 39 responses from employees and 13 responses from leaders. We imported the gathered data into IBM SPSS Statistic version 28.0.0 and Microsoft Office Excel version 2016, where we continued with the relevant analyses. The analysis was based on a correlational design. A major limitation of this type of statistical method is the sample size, namely, the size of the sample often determines the choice of statistical analysis. In our case, we collected a large enough sample that the statistical program showed the appropriate results. At the same time, our values do not contain outliers and a certain value does not differ significantly from the other values. By using commands to compare the mean values of individual variables, we came to reliable and representative results. Before this, we also performed a data reliability analysis for individual collected data and came to the results that there were no missing or corrupted values for any data collected using the LMX and linguistic intelligence questionnaires. At the same time, the validity and reliability of the results were confirmed using Cronbach’s alpha coefficient with a value of α = 0.831 for the LMX-7 questionnaire, and α = 0.617 for the linguistic intelligence questionnaire.

Then, we performed a correlation test between LMX-7 and the age and gender of the employees, and between linguistic intelligence and the age and gender of the leaders. The results showed that there was no statistical significance between the variables in the obtained data and results; so, we continued with the analysis and showed the age and gender of the respondents only in the descriptive method.

We used a correlational design to test the correlation between the leader’s linguistic intelligence and the quality of the LMX. We checked the quality of the LMX using the LMX-7 questionnaire and obtained the result that there was a high level of LMX quality in the organizations that were part of this study. Despite the fact that the leaders of the organizations practiced high-quality LMX, it is beneficial to recognize that there is still a possibility that employees falsely defined the variables when answering the LMX-7 questionnaire. This would certainly affect the results themselves, but given that the participants participated in the research voluntarily and anonymously, and that the LMX-7 questionnaire is considered to be the gold standard in the field of LMX with a great reliability rating, we consider the obtained results to be credible and representative.

Even though the results are representative and statistically significant, the results of the analysis do not apply to the entire population. The results obtained via the survey and questionnaire apply only to the selected sample included in the research. In order to obtain more thorough data on the quality of the LMX being dependent on the leader’s linguistic intelligence, a survey should be conducted on a larger scale and with a larger and more diverse sample.

Through data analysis, we were able to successfully verify the initial statement and we can now answer the thesis, which stated the following: “The leader’s linguistic intelligence is positively related to the quality of the leader–member exchange”. First, we used a questionnaire based on the leader’s linguistic intelligence to confirm that the respondents had this intelligence. Later, we used the LMX-7 questionnaire to confirm that LMX was practiced at a high level in the organizations in question.

Using the Pearson (r = 0.724; *p* = 0.005; α = 0.01; *p* < α) and Spearman (r = 0.580; *p* = 0.038; α = 0.05; *p* < α) correlation coefficient tests in SPSS, we found that LMX and linguistic intelligence have a positive high correlation. Continuing with linear regression analysis and the ANOVA test, the coefficient table showed that the *p* value was *p* = 0.005, which is lower than the predetermined level of significance of α = 0.05 (*p* < α). Thus, we can also conclude that the results are statistically significant and that the data provide sufficient evidence to confirm that the leader’s linguistic intelligence is highly positively related to the quality of the leader–member exchanges.

With this paper, we further contribute to the development and recognition of the potential of LMX implementation. With past research, authors have already focused on LMX and studied the connection with other areas such as workplace stress, job satisfaction and the emotional intelligence of a leader. With our study, we are not focusing on job satisfaction and the emotional intelligence of the leader, but on a leader and his content of linguistic intelligence, which is part of the multiple intelligences proposed by Gardner (1983, 2010).

Our field is more targeted and focused on the leader of the organization, the skills and intelligence that the leader has and how these skills are in correlation with the quality of the LMX. Despite the fact that the importance of a leader’s content of intelligence in general has already been theoretically defined and connected via case studies (Barbuto and Bugenhagen 2009; Gardner 2010; Fambro 2019; Palthe 2019), the influence and connection of a leader’s content of linguistic intelligence, with the quality of the LMX, according to descriptive and empirical scientific methods, have not been investigated, which was our motive for this study.

In this paper, we have considered only one part of LMX theory. However, there is much more to LMX theory that is worth researching and making known. Organizations are constantly and rapidly developing and adapting to the world around them. In order to survive, organizations are forced to look for alternative ways and approaches to leadership, and regardless of the weaknesses and potential dangers of leader–member exchanges, LMX theory is now considered to be innovative and effective (Mulligan et al. 2021).

### 4.1. Research Limitations

This study has some limitations. Despite the fact that we obtained representative results in our study, the sample on which we conducted the research is small due to the choice of purposive sampling; therefore, we encountered a limitation regarding the generalization of the results to the wider population. Working with smaller samples can quickly become a problem when analyzing the gathered data.

It is good to carry out research in the future based on a larger and more diverse sample, i.e., in larger organizations, with more organizations and among more leaders. The analysis should then be conducted individually between leaders and their respected employees. In our case, age and gender did not have statistical significance on the obtained data and results due to the small size of the sample; so, we were unable to demonstrate the influence of these variables on the quality of the LMX and the content of the leader’s linguistic intelligence. With larger and diverse samples and with a more rigorous set methodology and research approach, the obtained data and results could be generalized to a wider population and the study itself would have better external validity.

### 4.2. Future Research Proposals

Although we received a representative sample and the questionnaires had sufficient reliability, our findings apply only to our study and to the organizations that were included in said study. In order to generalize such findings to a wider population and have greater external validity, it is recommended that a similar study be conducted in the future, which would include a much larger and diverse sample of leaders and employees from organizations that practice LMX theory, and the methodology itself would be set more rigorously.

Future research should also look at the example of a leader with less developed linguistic intelligence and examine what impact this alone has on the quality of relationships between employees in the organization and the quality of the LMX itself. With a larger sample, we must also take into account the gender and age of both leaders and employees; namely, these variables also possibly have an influence on the quality of the LMX and the content of the leader’s linguistic intelligence, which is currently unknown. Further research would make an important contribution to this study and to the field of LMX theory. In this study, we have confirmed that a leader’s linguistic intelligence is highly positively related to the quality of the LMX. Based on other research, it has also been confirmed that employee commitment, job satisfaction and performance have a high positive connection with LMX, but we noted that further moderation and the influence of the leader’s linguistic intelligence on these mentioned factors have not yet been investigated. Further research on this part would make an important contribution to this study and to the field of LMX.

## 5. Conclusions

With this study, we have highlighted the importance of communication within the organization in general. In the case of LMX, communication is even more important because it is how a leader creates and maintains good interpersonal relationships, which in turn serve as a starting point for all other job-related satisfactions and processes. Accordingly, the leader must have a certain level of linguistic intelligence. Indeed, we have proven that there is a positive and high correlation between these two concepts.

Leader–member exchange has been present in the business environment for several decades, and much has been written and is already known about LMX theory. Yet, to date, no research has been found to determine the correlation between a leader’s linguistic intelligence and LMX. Previous studies have highlighted the importance of emotional intelligence, but communication was vastly mentioned by the authors as a very important part of practicing and implementing quality LMX for a better organizational climate, higher work engagement and greater commitment. In an ever-changing world, organizations are constantly facing external challenges and pressures. We cannot escape the changes, but we can manage them well with the LMX approach and its norms.

To ensure that the potential of LMX theory can be realized, it is necessary to be aware of all of the aspects that contribute to the quality implementation of LMX. Only then can we enjoy the benefits and advantages, and it all starts with the leader.

## Figures and Tables

**Table 1:** Case processing summary for LMX-7 questionnaire data.

		N	%
Cases	Valid	39	100.0
	Excluded ^a^	0	0.0
	Total	39	100.0
(**a**): Reliability statistics for LMX-7 questionnaire data			
**Cronbach’s Alpha**		**No. of Items**	
0.831		7	

^a^ Listwise deletion based on all variables in the procedure.

**Table 2:** Case processing summary for linguistic intelligence questionnaire data.

		N	%
Cases	Valid	13	100.0
	Excluded ^a^	0	0.0
	Total	13	100.0
(**a**): Reliability statistics for linguistic intelligence questionnaire data			
**Cronbach’s Alpha**		**No. of Items**	
0.617		10	

^a^ Listwise deletion based on all variables in the procedure.

**Table 3:** Average of sums for LMX.

Average of Sums		No. of Data	
29.02		39	
(**a**): Reliability Statistics for LMX			
**Cronbach’s Alpha**	**Cronbach’s Alpha Based on Standardized Items**		**No. of Items**
0.831	0.837		7

**Table 4:** Linguistic intelligence questionnaire means.

	Q1	Q2	Q3	Q4	Q5	Q6	Q7	Q8	Q9	Q10
Mean	4.15	4.08	4.15	3.85	3.54	4.15	3.92	3.54	4.46	4.46
Std. Error of Mean	0.222	0.137	0.191	0.154	0.268	0.191	0.211	0.144	0.144	0.183
(**a**): Linguistic intelligence questionnaire average of means										
**Average of Means**					**No. of Items**					
4.03					10					

**Table 5:** Reliability statistics for linguistic intelligence.

Cronbach’s Alpha	Cronbach’s Alpha Based on Standardized Items	No. of Items
0.617	0.604	10

**Table 6:** Descriptive statistics—employees’ gender.

		Frequency	Percent	Valid Percent	Cumulative Percent
Valid	Female	13	33.3	33.3	33.3
	Male	26	66.7	66.7	100.0
	Total	39	100.0	100.0	
(**a**): Descriptive statistics—employees’ age					
		**Frequency**	**Percent**	**Valid Percent**	**Cumulative Percent**
Valid	29	4	10.3	10.3	10.3
	30	2	5.1	5.1	15.4
	31	3	7.7	7.7	23.1
	32	5	12.8	12.8	35.9
	34	3	7.7	7.7	43.6
	35	2	5.1	5.1	48.7
	36	11	28.2	28.2	76.9
	37	1	2.6	2.6	79.5
	38	3	7.7	7.7	87.2
	39	1	2.6	2.6	89.7
	40	1	2.6	2.6	92.3
	41	1	2.6	2.6	94.9
	42	1	2.6	2.6	97.4
	43	1	2.6	2.6	100.0
	Total	39	100.0	100.0	
(**b**): Statistical significance of employees’ age and gender on LMX-7 questionnaire data					
			**LMX_7**	**Employees—Please Fill in Your Gender.**	**Employees—Please Fill in Your Age.**
LMX_7		Pearson Correlation	1	0.082	0.257
		Sig. (2-tailed)		0.622	0.114
		N	39	39	39
Employees—Please fill in your gender.		Pearson Correlation	0.082	1	0.149
		Sig. (2-tailed)	0.622		0.365
		N	39	39	39
Employees—Please fill in your age.		Pearson Correlation	0.257	0.149	1
		Sig. (2-tailed)	0.114	0.365	
		N	39	39	39

**Table 7:** Descriptive statistics—leaders’ gender.

		Frequency	Percent	Valid Percent	Cumulative Percent
Valid	Female	4	30.8	30.8	30.8
	Male	9	69.2	69.2	100.0
	Total	13	100.0	100.0	
(**a**): Descriptive statistics—leaders’ age.					
		**Frequency**	**Percent**	**Valid Percent**	**Cumulative Percent**
Valid	26	1	2.6	2.6	69.2
	29	1	2.6	2.6	71.8
	31	1	2.6	2.6	74.4
	32	1	2.6	2.6	76.9
	34	2	5.1	5.1	82.1
	39	1	2.6	2.6	84.6
	41	1	2.6	2.6	87.2
	43	1	2.6	2.6	89.7
	54	1	2.6	2.6	92.3
	56	1	2.6	2.6	94.9
	57	1	2.6	2.6	97.4
	58	1	2.6	2.6	100.0
	Total	13	100.0	100.0	
(**b**): Statistical significance of leaders’ age and gender on linguistic intelligence questionnaire data.					
			**Linguistic Intelligence**	**Leader—Please Fill in Your Gender.**	**Leader—Please Fill in Your Age.**
Linguistic intelligence		Pearson Correlation	1	−0.095	0.322
		Sig. (2-tailed)		0.757	0.283
		N	13	13	13
Leader—Please fill in your gender.		Pearson Correlation	−0.095	1	−0.236
		Sig. (2-tailed)	0.757		0.437
		N	13	13	13
Leader—Please fill in your age.		Pearson Correlation	0.322	−0.236	1
		Sig. (2-tailed)	0.283	0.437	
		N	13	13	13

**Table 8:** Pearson correlation coefficient.

		LMX	Linguistic Intelligence
LMX	Pearson Correlation	1	0.724 **
	Sig. (2-tailed)		0.005
	n	39	13
Linguistic Intelligence	Pearson Correlation	0.724 **	1
	Sig. (2-tailed)	0.005	
	n	13	13

** Correlation is significant at the 0.01 level (2-tailed).

**Table 9:** Spearman’s correlation coefficient.

			LMX	Linguistic Intelligence
Spearman’s rho	LMX	Correlation coefficient	1.000	0.580 *
		Sig. (2-tailed)		0.038
		n	39	13
	Linguistic intelligence	Correlation coefficient	0.580 *	1.000
		Sig. (2-tailed)	0.038	
		n	13	13

* Correlation is significant at the 0.05 level (2-tailed).

**Table 10:** Model summary.

Model	R	R Square	Adjusted R Square	Std. Error of the Estimate	Change Statistics				
					R Square Change	F Change	df1	df2	Sig. F Change
1	0.724 ^a^	0.524	0.481	1.36802	0.524	12.100	1	11	0.005

^a^ Predictors: (constant) linguistic intelligence.

**Table 11:** ANOVA ^a^.

Model		Sum of Squares	df	Mean Square	F	Sig.
1	Regression	22.644	1	22.644	12.100	0.005 ^b^
	Residual	20.586	11	1.871		
	Total	43.231	12			

^a^ Dependent variable: LMX_7. ^b^ Predictors: (constant), linguistic intelligence.

**Table 12:** Coefficients ^a^.

Model		Unstandardized Coefficients		Standardized Coefficients	t	Sig.	95.0% Confidence Interval for B	
		B	Std. Error	Beta			Lower Bound	Upper Bound
1	(Constant)	12.290	4.951		2.482	0.030	1.392	23.187
	Linguistic intelligence	0.426	0.122	0.724	3.478	0.005	0.156	0.696

^a^ Dependent variable: LMX_7.

## Data Availability

The data presented in this study are available from T.R. upon request.

## Appendix A

LMX-7 questionnaire:

1. Kako pogosto se zavedate, kje stojite s svojo vodjo in kako pogosto se zavedate, kako zadovoljen je vaš vodja s tem, kar počnete?
2. Kako dobro vaš vodja razume vaše službene težave in potrebe?
3. Kako dobro vaš vodja pozna vaš potencial?
4. Ne glede na to, koliko formalne avtoritete ima vaš vodja glede na položaj, kakšna je verjetnost da bi vaš vodja uporabil svojo moč, da vam pomaga pri reševanju težav pri vašem delu?
5. Ponovno, ne glede na formalno avtoriteto, ki jo ima vaš vodja, kakšna je verjetnost, da bi vas na svoj račun razbremenil ali »rešil«?
6. V svojo vodjo imam dovolj zaupanja, da bi ga branil in utemeljil svojo odločitev, če pri tem vodja ne bi bil prisoten.
7. Kako bi opisali svoj delovni odnos z vodjo?

## Appendix B

Linguistic intelligence questionnaire:

1. Že od malih nog zelo uživam v branju knjig, revij ali drugih spisov.
2. Naučim se pomena glasov, ki so zame novi.
3. Ugotavljam razlike med besedami s podobnim pomenom.
4. Moji prijatelji pravijo, da znam pojasnjevati različne teme.
5. Pišem kratke zgodbe, poezijo ali članke.
6. Ko govorim ali pišem, uporabljam različne besede.
7. Najraje imam izpite ali teste, kjer lahko svoje odgovore razvijem v pisni obliki.
8. Dobro si zapomnim dolge sezname besed.
9. Ko pišem sestavek, izberem prave in natančne besede.
10. Ko pišem o temi, razmišljam o vrstnem redu, v katerem naj besede sledijo.
